# Flexible automation with compact NMR spectroscopy for continuous production of pharmaceuticals

**DOI:** 10.1007/s00216-019-01752-y

**Published:** 2019-03-23

**Authors:** Simon Kern, Lukas Wander, Klas Meyer, Svetlana Guhl, Anwesh Reddy Gottu Mukkula, Manuel Holtkamp, Malte Salge, Christoph Fleischer, Nils Weber, Rudibert King, Sebastian Engell, Andrea Paul, Manuel Pereira Remelhe, Michael Maiwald

**Affiliations:** 1 Bundesanstalt für Materialforschung und -prüfung (BAM), Richard-Willstätter-Str. 11, 12489 Berlin, Germany; 2 Department Measurement and Control, Institute of Process Engineering, Berlin University of Technology, Hardenbergstr. 36a, 10623 Berlin, Germany; 3 Department of Biochemical and Chemical Engineering, Process Dynamics and Operations Group, Dortmund University of Technology, Emil-Figge-Str. 70, 44227 Dortmund, Germany; 4 INVITE GmbH, CHEMPARK, 51368 Leverkusen, Germany; 5 Bayer AG, Kaiser-Wilhelm-Allee, 51368 Leverkusen, Germany

**Keywords:** NMR spectroscopy, NIR spectroscopy, Real-time process monitoring, Real-time quality control, Continuous processes

## Abstract

Modular plants using intensified continuous processes represent an appealing concept for the production of pharmaceuticals. It can improve quality, safety, sustainability, and profitability compared to batch processes; besides, it enables plug-and-produce reconfiguration for fast product changes. To facilitate this flexibility by real-time quality control, we developed a solution that can be adapted quickly to new processes and is based on a compact nuclear magnetic resonance (NMR) spectrometer. The NMR sensor is a benchtop device enhanced to the requirements of automated chemical production including robust evaluation of sensor data. Beyond monitoring the product quality, online NMR data was used in a new iterative optimization approach to maximize the plant profit and served as a reliable reference for the calibration of a near-infrared (NIR) spectrometer. The overall approach was demonstrated on a commercial-scale pilot plant using a metal-organic reaction with pharmaceutical relevance.

**Electronic supplementary material:**

The online version of this article (10.1007/s00216-019-01752-y) contains supplementary material, which is available to authorized users.

**Figure:**
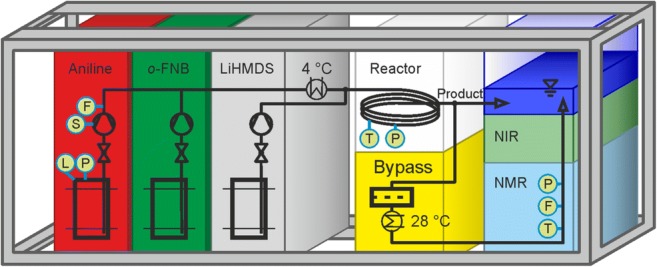
Graphical abstract

## Introduction

The pharmaceutical industry is making considerable efforts to establish continuous manufacturing of active pharmaceutical ingredients (API) as an alternative to batch production. A study released by representatives of ten prominent pharmaceutical companies demonstrates the value of intensified continuous manufacturing with regard to improved quality, safety, sustainability, throughput time, speed of implementation, and profitability [1–3]. The combination of continuous processes with a modular plant concept supporting plug-and-produce reconfiguration enables efficient and flexible production of different substances using standardized modular equipment [4, 5].

Online quality monitoring and model-based control of critical quality attributes (CQAs) are required to ensure the desired product quality and to run a continuous process in an optimal way [6]. The effort to develop such integrated control solutions slows down the implementation of new continuous API processes considerably and hampers flexibility when making many different products. Process analytical methods such as online near infrared (NIR), UV/VIS, or Raman spectroscopy are typically employed for online quality monitoring [7–9]. The calibration of such instruments is usually expensive and time consuming. A considerable set of different medium samples has to be obtained and a suitable reference analytical method has to be developed to determine the compositions of the samples. Furthermore, a calibration model correlating spectral sensor data to the corresponding chemical compositions has to be fitted, validated, and maintained.

Seeking for appropriate process analytical methods, quantitative NMR spectroscopy was considered in this study, which features a high linearity between absolute peak areas in the spectra and the concentrations of analyte molecules in the samples. This makes it an absolute analytical comparison method being strictly independent on the sample matrix, e.g., solvent effects. NMR spectroscopy provides information about the structure of the molecules in the sample as well as quantitative information. ^1^H NMR spectroscopy provides several concentration readings as well as structural information per minute. The high rate of data points helps in understanding the dynamics of the investigated pilot plant. Most NMR spectrometers use cryo-cooled superconductors to establish a very strong magnetic field (> 5 T), but they are not suitable for process applications because of the need for cryogenic liquids, the high operational costs, and the large size of these devices. Nowadays, compact NMR systems are available using permanent magnets with a lower magnetic flux density (i.e., 1 T) and sufficient field homogeneity below 1 Hz. These devices are small, relatively inexpensive, and very appealing for process applications [10, 11]. Automated systems including compact NMR spectrometers have been reported in literature lately focusing on reaction monitoring [12], self-optimizing reactor systems [13], or in combination with an organic synthesis robot for the prediction of reactivities [14]. However, these prototype devices and commercially available instruments are primarily intended for laboratory use.

In this contribution, we present a real-time quality control solution that can be adapted quickly to new processes. It includes a compact NMR spectrometer for online quality monitoring and a new data and model-based process control approach. The integrated solution was developed for a lithiation reaction running on a commercial-scale modular pilot plant and it was tested under industrial conditions within the European Union’s Horizon 2020 project CONSENS (Integrated Control and Sensing for Sustainable Operation of Flexible Intensified Processes, 2015–2017). A benchtop NMR spectrometer was converted into a smart, compact (57 × 57 × 85 cm), portable process analytical sensor which easily benefits the modular plant concept. It rapidly and non-invasively measures the chemical composition with minor calibration effort and without the need for sample preparation, or deuterated solvents. The analytes are quantified with modular, physically motivated models. These models can be adapted to new substances solely by the use of their corresponding pure component spectra. Beyond that, we comprehensively calibrated an NIR spectrometer based on real NMR process data for the first time within an industrial plant. The advantage of this approach allowed us to include reaction components, which are typically not accessible for offline reference analytics. This comprises intermediates usually depleted after quenching of the technical samples and additional components such as contaminants and side products. The iterative control approach leads to the economically optimal operation of the plant even though the process model is not accurate.

## Materials and methods

### Experimental setup in pilot scale

The pilot plant considered here was built in a previous research project to demonstrate the feasibility of mobile and modular intensified continuous production [15]. It is characterized by a strictly modular architecture based on a standard 20-ft shipping container with the dimensions 6.0 m × 2.4 m × 2.6 m (L × W × H). The plant was designed to produce an API intermediate with a capacity of several tons per year including two reaction steps and two purification steps. In this study, only the first reaction unit is considered which is an air-cooled tubular reactor. A highly exothermal metal-organic reaction is used in which two aromatic compounds, aniline and 1-fluoro-2-nitrobenzene (*o*-FNB), are coupled using lithium hexamethyldisilazane (LiHMDS) as a base yielding lithium-2-nitrodiphenylamine (Li-NDPA) and lithium fluoride.

**Fig. 1:**
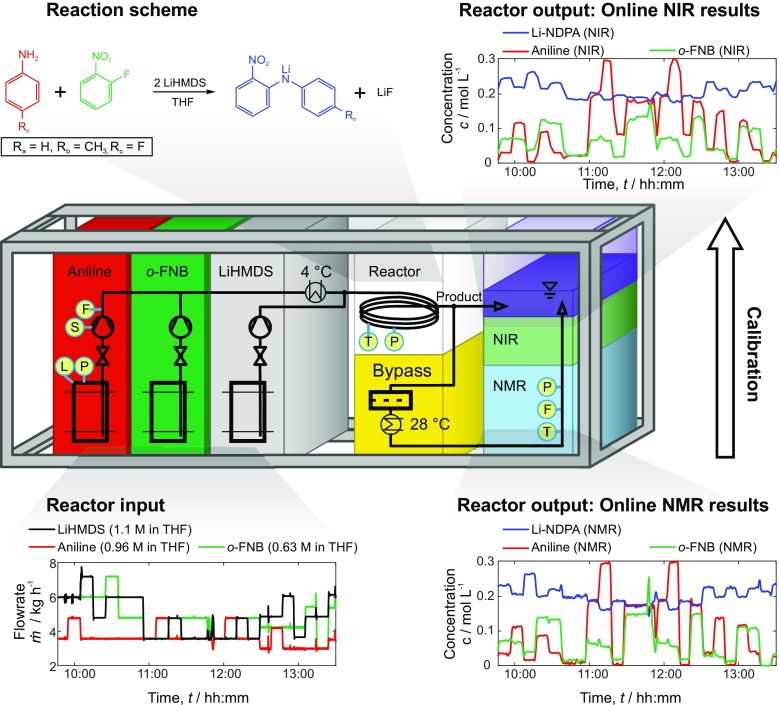
Schematic visualization of the modular pilot plant for the production of an API intermediate. The modular plant is comprised of dosing units feeding the reactants aniline, *o*-FNB, and LiHMDS into a tubular reactor. A bypass is used to route a filtered fraction of the product stream through an online NMR and NIR sensor. Online NMR spectroscopy was used for rapid calibration of NIR data using online reference values. Relevant locations of measurements are indicated as L = level, P = pressure, S = speed of rotation, F = flow, and T = temperature

The setup of the relevant parts of the plant and the reaction scheme are illustrated in Fig. 1. Three separate dosing units provide the reactants each dissolved in tetrahydrofuran (THF). After pre-mixing of anline and *o*-FNB, cooling to 4 °C is necessary to avoid THF from boiling in the tubular reactor. At least 2 mol of LiHMDS has to be applied to 1 mol aniline and 1 mol *o*-FNB. Due to the rather loose raw material specification of the lithium base and side reactions with residual moisture, LiHMDS is used in excess. In this study, the initial stoichiometric factor of the lithium base was set to 2.14. The flow rates for the THF solutions of *o*-FNB (5.60 kg h^−1^), aniline (3.68 kg h^−1^) and LiHMDS (6.89 kg h^−1^) were selected accordingly, assuming concentrations 0.63, 0.96, and 1.10 mol L^−1^ of the analytes, respectively. These concentrations may vary during production when the tanks of the dosing units are filled up with new batches of raw material solutions. Hence, these flow rates are used for start-up and are adjusted during production by the control algorithm to compensate variations of feed compositions and temperatures, and to optimize the operation of the plant. The pilot plant has to be considered as a hazardous area prone to explosive atmospheres, due to the use of THF and other organic substances. Thus, all devices used in the plant, including the online NMR sensor, are required to meet explosion protection regulations for zone 1 according to ATEX (European Directives for controlling explosive atmospheres).

During the start-up procedure on each production day, the pure *o*-FNB solution was pumped through the analytic modules for one-point calibration of the NMR spectrometer. Subsequently, initial flowrates based on stochiometric assumptions were set manually. The iterative optimization scheme based on online NMR data was initialized subsequently. It performed step changes in the raw material flow rates to identify the plant optimum while the process was running. After each iteration step, the flow rates were kept constant for about 10 min to reach steady-state concentrations. After commissioning the plant, a total of 4 days of plant operation, spread over a period of 3 weeks, yielded a total of 40 h of process data.

### Chemicals

Lithium hexamethyldisilazane (LiHMDS), typically in the form of 1.1 mol L^−1^ solution in THF/ethylbenzene was purchased from Albemarle Corporation (Charlotte, NC, USA). The ethylbenzene content within the LiHMDS solution was specified maximal 9 wt%. The raw materials aniline, *o*-FNB (both AK Scientific Inc., Union City, USA), and THF (Brenntag GmbH, Essen, Germany) were purchased in technical grade and used without prior purification.

### Online low-field NMR spectroscopy

The NMR instrument (Spinsolve Proton, Magritek, Aachen, Germany), operating at 43.32 MHz ^1^H frequency, comes with 5-mm ID bore for standard NMR tubes at a magnet temperature of 28.5 °C. The system was operated with a Windows computer and was triggered via XML commands. For the studies presented here, a 5-mm glass flow cell (ID = 4 mm) was used. Online proton spectra were acquired with single scans, 90° pulse, 6.5-s acquisition time, and 8.5-s recycle delay yielding a repetition time of 15 s.

NMR data was saved locally in binary files and processed in real time via MATLAB (R2017a, The MathWorks, Natick, MA, USA) using automatic folder monitoring. The free induction decay (FID) was zero filled to 64 k data points and subsequently apodized by exponential multiplication with a line broadening factor of 0.5 Hz. After Fourier transformation, spectra were treated with automated data preparation algorithms. These were baseline correction [16], phasing [17], and spectral alignment to a reference spectrum (neat THF) using the icoshift algorithm [18]. Concentration values were calculated based on the processed spectra using indirect hard modeling (IHM). For automation purposes, component fitting and calculation of concentration values during real-time optimization experiments at the pilot plant was implemented in MATLAB. Reference concentration values for calibrating the NIR spectrometer were calculated offline with the PEAXACT software (SPACT GmbH, Aachen, Germany). Small differences exist between the MATLAB and PEAXACT (not shown) approach.

Model parameters for IHM were adopted from previous studies of the same reaction system at lab-scale [19]. For the absolute quantification method, the use of a concentration conversion factor *ξ* was applied to convert signal areas to molar concentration *c*_*i*_. *ξ* was determined via one-point calibration obtained from the known concentration of the *o*-FNB solution using Eq. 1:

1 $$ {c}_i=\upxi \bullet \frac{A_i}{\upnu_i} $$

where *ν* is the number of nuclei and *A* is the corresponding absolute integral.

### Online NIR spectroscopy

FT-NIR absorbance spectra were measured with a fiber optic transmission probe (Knauer A4081) using a Matrix-F spectrometer (Bruker Optik GmbH, Ettlingen, Germany). 64 consecutive sample scans were accumulated in the spectral range of 4000–12,000 cm^−1^ with a spectral resolution of 8 cm^−1^. Zero filling with factor 2 and apodization using a Blackman-Harris-3-Term function yielded a total of 2074 data points per spectrum. Measurement intervals were set to 160 s. Data evaluation including preprocessing and multivariate calibrations was performed using The Unscrambler X, Version 10.5 (CAMO Software, Oslo, Norway).

### Field integration of analytical instrumentations

The compact NMR spectrometer was integrated into an online sensor unit, equipped with a pressurized enclosure with nitrogen purge (see Electronic Supplementary Material (ESM) Fig. S2 and S3). The resulting sensor module was certified by a notified body according to ATEX regulations which allows the usage of the sensor in the hazardous area of the pilot plant. The product flow through the NMR instrument was controlled inside the module to 1.5 mL min^−1^ to assure complete pre-magnetization of the sample flow. A detailed piping and instrumentation diagram (P&ID) and the spatial arrangement depicted in Figs. S2 and S3 (see ESM) show the working principle of the whole NMR sensor. The NMR unit was fully automated, including acquisition, processing, and communication of data. The concentration values of the selected substances were transmitted to the distributed control system through the standardized communication protocol OPC Unified Architecture.

**Fig. 2:**
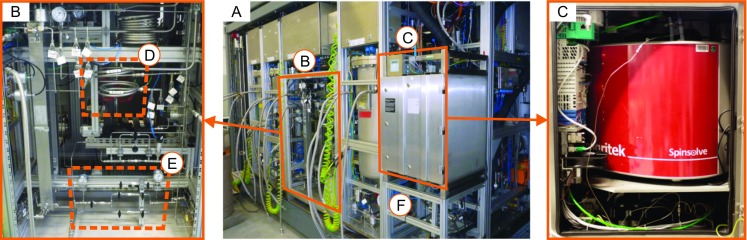
Labeled photograph of modular pilot plant (**a**) for the continuous 2-nitrodiphenylamine synthesis. (**b**) Close-up example of tubular reactor (**d**, ID = 12.4 mm) and the filter section (**e**). (**c**) Photograph of the integrated compact NMR spectrometer (43 MHz) with ATEX certified pressurized housing for online concentration measurements. (**f**) indicates the location of the NIR flow cell

A small fraction of the product stream, approximately 1% of total flow, was separated in a bypass for online analytics with NMR (Fig. 2, (C)) and NIR spectroscopy (Fig. 2, (F)). The flow rate through the online analytical modules was induced by a pressure drop of 3 bars from the main product stream. Since particle formation from water residue precipitation of LiF and LiOH is a major source of clogging in small capillaries of the bypass (ID = 1 mm), a filter module for the reduction of particle loads was implemented. A combination of redundant 90- and 60-μm stainless steel inline filters (Swagelok) has evolved as a robust arrangement to prevent clogging in the bypass (see Fig. 1 and S4).

NMR measurements were taken in 15-s intervals, while NIR spectra were recorded in 160-s intervals. NIR measurements were recorded in transmission mode in close proximity to the NMR device, before the product left the pilot plant.

### Real-time optimization experiments

In model-based optimization, exact non-linear process models are needed to determine the real optimum operating point of a plant. However, most often a lot of work is needed to describe all effects of the process behavior precisely enough. In this case, it is very costly to determine the reaction kinetics, since the reaction is very fast, and it is assumed that mixing effects in the tubular reactor influence the conversion considerably. Instead, a simple control model was built based on the stoichiometry of the reaction system and using rough estimates of the kinetic parameters based on chemical understanding. Due to the fast reaction and the short residence time in the reactor, the plant quickly reaches a steady state after changes of the feed rates. Therefore, an iterative steady-state optimization was implemented. The objective function of the optimization problem considers the prices and the mass flows of, the raw materials fed to the reactor and the product obtained at the outlet according to Eq. 2.

2 $$ \mathrm{Profit}={w}_4\bullet {M}_{\mathrm{Li}-\mathrm{NDPA}}\bullet {c}_{\mathrm{Li}-\mathrm{NDPA}}/{\rho}_{\mathrm{Mixture}}\bullet \sum \limits_{i=1}^3{u}_i-\sum \limits_{i=1}^3{w}_i{u}_i, $$

where *u*_1_, *u*_2_, and *u*_3_ are the feed flowrates of the reactants aniline, LiHMDS, and *o*-FNB in kg h^−1^, and *M*_Li-NDPA_ as well as *c*_Li-NDPA_ are the molar mass in kg mol^−1^ and the measured concentration in mol m^−3^ of the product Li-NDPA. Weights for each term in the profit function (*w*_1_–*w*_4_) reflect the relative costs of the reactants and the product and were set to 10,000, 25,000, 12,000, and 450,000 kg^−1^, respectively. The density of the reaction mixture (*ρ*_Mixture_) was set to 900 kg m^−3^.

In contrast to the traditional non-linear process modeling approach, no estimation of model parameters was performed, but the optimization problem itself was adapted by introducing correction terms, the so-called modifiers [20]. These modifiers describe the differences between the observed stationary behavior of the plant and its model and of the gradients of the cost function and of the constraints with respect to the manipulated variables. In this manner, the necessary conditions for optimality of the true plant operation are satisfied upon convergence. To use this kind of correction without identifying model parameters was first proposed by Tatjewski [21] and later extended to include the handling of constraints [22]. The critical element of this iterative optimization algorithm is the computation of the gradients from the plan measurements. To compute the gradients from finite differences is vulnerable to measurement noise and can lead to erratic control moves. A recently proposed modifier adaptation with quadratic approximation (MAWQA), in which the iterative gradient correction [22] is combined with elements from derivative-free optimization (DFO) to estimate the plant gradients [23], was adopted to the given optimization problem.

## Results and discussion

### Flexible data evaluation approach for NMR spectra

**Fig. 3:**
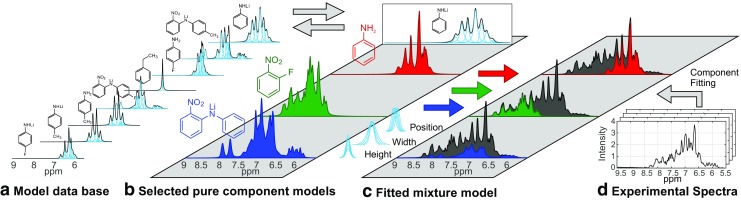
Indirect Hard Modeling (IHM) workflow for quantitative evaluation of measured NMR spectra (**d**) by building a mixture model (**c**). Relevant pure component models (**b**) for each process can be selected from pure a component model database (**a**) and employed together with model constraints. Cyan lines represent peak functions of each spectral model

Peak signals within the spectra of compact NMR devices tend to spread and overlap due to the weaker magnetic field of permanent magnets compared to instruments with higher magnetic field strengths. Since the individual peak areas are not directly accessible, deriving chemical compositions from such NMR spectra is challenging. A spectral deconvolution method was applied that relies solely on pure component NMR spectra of the substances expected in the chemical process (reactants, products, and major impurities). The chemical composition of an unknown mixture is determined by fitting a superposition of the pure component spectra to the measured NMR spectrum of the mixture (Fig. 3). This component fitting is realized by an advanced line fitting approach called IHM in which the pure component spectra are modeled by sums of basic peak functions (i.e., Lorentzian-Gaussian functions represented by Pseudo-Voigt functions). The IHM algorithm adjusts heights, widths, and positions of the basic peak functions within predefined model constraints to minimize the residuals between measured data and the mixture model, but it maintains the peak area ratios in each pure component model for physical plausibility [24, 25]. The compositions can then be derived from the resulting areas of the substance models. IHM applied to complex NMR spectra enables a calibration-free access to molar ratios and provides absolute concentrations with low calibration effort (one-point calibration) [26].

Prior to deployment at the modular plant, the proof of principle for the NMR sensor and of the flexibility of the data evaluation concept was validated in the laboratory for various sets of starting materials and the resulting products. The functional groups R of the aromatic amine within the metal-organic reaction (Fig. 1) comprised aniline (R = H), 4-flouroaniline (R = F), and toluidine (R = CH_3_). Initially, a spectral library of various analytes for the investigated reaction system was created, including pure component spectra measured offline in standard NMR tubes (ESM Fig. S1) and their derived spectral models (Fig. 3, (**a**)). For each experiment within the laboratory setup, relevant pure component models were selected (Fig. 3, (**b**)). The experimental setup of this study as well as model constraints of IHM for the reaction system with R = H were recently published [19]. The investigation of various starting materials revealed that model constraints from [19] could be universally applied for data evaluation in all three cases. Root-mean-square errors (RMSE) for the concentration measurements of each reaction system are depicted in Table 1. RMSE values in the range of 5–16 mmol L^−1^ were achieved. Online high-field NMR spectroscopy (500 MHz) served as a reference method for validation since metal-organic reactants are problematic to analyze via offline sampling due to their sensitivity to air and moisture.

**Table 1:** RMSE values for quantitative analysis of NMR spectra (43 MHz) using Indirect Hard Modeling (IHM) for the synthesis of products NDPA (2-nitrodiphenylamine), MNDPA (2-nitro-4′-methyldiphenylamine), and FNDPA (2-nitro-4′-fluorodiphenylamine). Depending on the desired product, the applied aromatic amine for the reaction was aniline, *p*-toluidine, or *p*-fluoroaniline

Reaction system		NDPA	MNDPA	FNDPA
Process mode		Batch	Continuous	Continuous
Number of NMR spectra		529	1395	50
RMSE values				
Product	/mmol L^−1^	12	12	4
*o*-FNB	/mmol L^−1^	14	5	9
Arom. amine	/mmol L^−1^	16	12	6
Li-arom. amine	/mmol L^−1^	–	10	10

### Iterative optimization of the plant performance

The goal of the control solution was to guarantee a consistent product quality, to make sure that the safety constraints of the process are not violated, and to drive the plant to its economically optimal operating point at the same time. This is not trivial, because there are external disturbances that need to be compensated. For instance, when a feed buffer is refilled with a new batch of raw material, the composition of the respective feed stream may shift. In addition, temperature variations in the cooling system and in the environment can influence the process. The implemented control solution reads temperature measurements and the chemical composition from the NMR sensor and manipulates the set points of the local flow controllers of the dosing units.

**Fig. 4:**
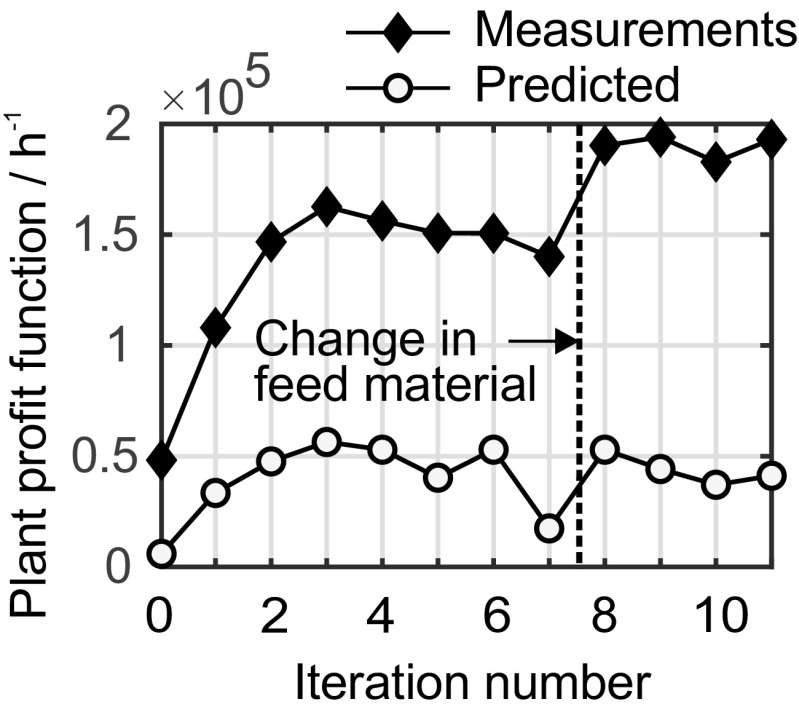
Plant profit in cost units per hour over MAWQA iterations performed at the modular pilot plant on day 4 (17.10.2017). *Diamonds*: real plant profit computed from the NMR measurements. *Circles*: plant profit computed from the nominal model, which has both structural and parametric plant model mismatch. *Dashed line*: the LiHMDS feed module was filled up with a new batch

In order to validate the performance of the iterative optimization scheme, it was started far from the plant optimum. To begin with, a feed flowrate of 3.58 kg h^−1^ for aniline, LiHMDS, and *o*-FNB was used. Upon initiation, several probing moves were performed to compute the plant gradients. Subsequently, optimization by MAWQA was started. The input moves generated by the iterative optimization scheme on day 4 of the experiments and the corresponding plant measurements are shown in Fig. S13 (see ESM), as the most prominent example. The plant profit during iterative optimization using the NMR measurements was significantly improved, as shown in Fig. 4. Changing the feed material caused an unknown change to the feed concentration of LiHMDS. The algorithm then further improved the plant profit. This improvement was not predicted by the nominal plant model.

### Novel approach for NIR calibration

Near-infrared (NIR), Raman, and mid-infrared (MIR or IR) spectroscopy are increasingly used for online monitoring of product quality in the industry [7, 27]. NIR can be easily implemented in industrial environments despite additional explosion protection requirements. Most complex analytical sensors do not yet fulfill safety integrity level (SIL) demands. Similar chemical information simultaneously provided by NIR and NMR spectroscopy might improve their acceptance in safety functions due to the redundancy they offer. In complex mixtures, such as the lithiation reaction, calibration of NIR instruments often relies on a multivariate approach. To cover all possible chemical states of the reaction mixture in a calibration model, design of experiments is typically applied. For each variation in the chemical structure of raw materials, near-infrared spectra combined with reference data is required to develop new calibration models. Those reference values are conventionally obtained from labor-intensive laboratory experiments and offline analytics (e.g., HPLC or GC-MS), which requires sampling from the continuous production stream. Since combined sampling errors are usually one or two orders of magnitude larger than the analytical uncertainty, the dominant impact on data quality is the sampling process [28]. Using online analytics for calibration also grants access to formerly (via offline methods) inaccessible intermediates (i.e., Li-aniline), which are vanishing during sampling or quenching of the reaction mixture.

By using the aforementioned calibration workflows for optical sensors, calibration model development for spectroscopic data can be time-consuming and significantly slows down implementation of the final process. Recent cost decreases and performance improvements will increase the number of NIR sensors in chemical plants [29], demanding innovative alternatives to reduce setup times and analytical lead time. We propose a new calibration approach of NIR sensors using concentration values provided by an online NMR module as a reference.

#### Selection of reference values

**Fig. 5:**
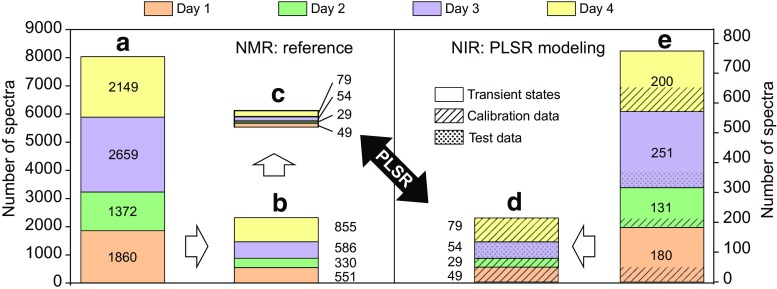
Visualization of the data selection prior to PLSR modeling. A total of 8040 NMR and 762 NIR spectra were recorded during four runs at the pilot plant on four different days (**a**, **e**). NMR spectra measured during steady concentration states (**b**) were extracted. Due to shorter measurement intervals, more NMR spectra than NIR spectra were available. By a nearest-neighbor approach, the corresponding NMR and NIR spectra during steady states were matched (**c**, **d**). Based on the two data sets (**c**) and (**d**), PLSR models were developed. NIR data acquired during steady states (**d**) were therefore split into a calibration and a test set. The remaining NIR spectra during transient states were used as an additional test set

The difference of delay time for both sensors (2 min) was compensated for prior to correlating the measurements, according to the mean residence times. For reliable calibration of the NIR sensor with NMR data, only measurements during steady-state phases were considered. Steady-state conditions of the system were important to prevent reference concentrations from being affected by concentration gradients moving through both spectrometers while acquiring spectra. For each analyte, a moving linear fit was calculated from 11 consecutive online NMR concentration values. Linear fit results with both a slope below 0.1 mol h^−1^ and standard deviation below 0.01 mol L^−1^ among the data points were classified as steady states. If these conditions were not fulfilled, the respective spectrum was classified as a transient state. Additionally, during steady states, NMR concentration values were smoothed using a moving average with a block size of six data points. Figure 5 shows the reference data selection process in detail. The 211 NIR spectra available during the steady-state condition were split into a calibration set (day 1, 2, and 4) and a test set (day 3), which yielded 157 and 54 spectra, respectively. An even distribution of the assigned reference concentration values within the calibration set and the test set was maintained (see ESM Fig. S6).

#### Exploratory data analysis

The recorded NIR spectra were characterized by three different spectral regions (ESM Fig. S5 A). At high wavenumbers (12,000–8975 cm^−1^), strong absorption and noise dominate spectra with high product concentrations, and strong purple color can be observed in the product solution. Absorption of high energies in NIR spectra is often present in colored solutions as a result of electronic transition. In addition, particles are formed during Li-NDPA formation due to the low solubility of LiF in THF. Despite the presence of a filter section, small particles (< 30 μm) are a possible source of light scattering. Additional noise is present at the lower energy limit of the spectra (4611–4000 cm^−1^). The central region (8957–4611 cm^−1^) is feasible for quantitative analysis.

#### Multivariate data analysis

For quantitative prediction of each of the four analytes (aniline, *o*-FNB, Li-NDPA, and Li-aniline), partial least squares regression (PLSR) was performed. At first, PLSR models for pooled spectral data of runs occurring on day 1, day 2, and day 4 were optimized by cross-validation over 20 randomly chosen segments. PLSR models were calculated for all analytes separately using the resulting RMSE of cross-validation (cf. Table 2) as a figure of merit for model optimization. Among the mathematical pretreatments tested [30], the best results were obtained with standard normal variate (SNV) transformed spectra in the range of 8957–4611 cm^−1^ as an initial step for aniline, Li-NDPA, and the lithiated aniline species. For *o*-FNB, a baseline correction in the range 6307–5995 cm^−1^ using a third-order polynomial yielded best results. For aniline and Li-aniline, the performance of the PLSR model was further improved by selecting characteristic absorption features followed by an additional baseline correction by subtracting either a second-order polynomial or a constant offset (see ESM Fig. S5 and Table S1). Unlike the three analytes mentioned, for Li-NDPA, the entire spectral range was evaluated.

**Table 2:** RMSE values from the quantitative analysis of NIR spectra by PLSR. Models were optimized based on RMSE values of (*i*) cross-validation (CV) over 20 randomly chosen segments, (*ii*) systematic CV and (*iii*) prediction of the test set. The optimal number of factors of each approach is indicated in parenthesis

Substance	Factors chosen	Random CV/mmol L^−1^	Systematic CV/mmol L^−1^	Test set/mmol L^−1^
Aniline	1	12 (1)	14 (1)	10 (1)
*o*-FNB	3	12 (3)	16 (3)	12 (3)
Li-NDPA	5	9 (5)	16 (5)	14 (5)
Li-aniline	4	7 (4)	9 (2)	7 (4)

The optimized PLSR models were validated using the remaining data set (day 3) which was not part of the calibration data. The resulting RMSE for the test set were close to those obtained by random cross-validation (Table 2). Moreover, a systematic cross-validation over all four batches was performed yielding comparable RMSE values indicating high reproducibility among the runs tested.

#### Quantitative results

**Fig. 6:**
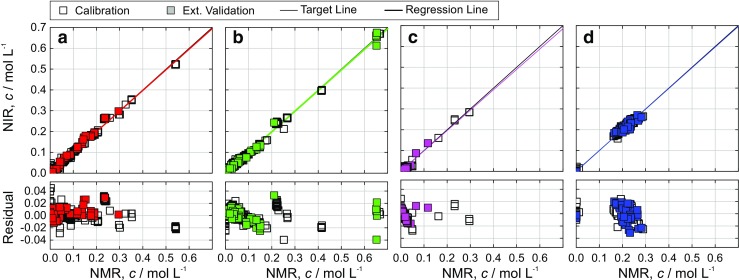
Calibration results of NIR data within the continuous production of 2-nitrodiphenylamine (NDPA). The parity plots show the predicted concentration values versus the online reference method (43-MHz proton NMR spectra). The test data set yielded RMSE values of 10, 12, 7, and 14 mmol L^−1^, respectively, for aniline (**a**), *o*-FNB (**a**), Li-aniline (**c**), and the product Li-NDPA (**d**), cf. Table 2

The prediction of analytes within the steady states based on the established PLSR models for NIR exhibited a respectable predictive performance (Fig. 6). Besides the product Li-NDPA, the intermediate lithiated form of aniline, Li-aniline could successfully be detected by NIR spectroscopy. NIR spectra recorded during transient states were used for the prediction of aniline, *o*-FNB, Li-aniline, and Li-NDPA (ESM Figs. S7–S14). With minor exceptions, the predicted concentration values presented the course of the reaction very well when compared to NMR data. Small deviations between NIR- and NMR-derived concentrations can be explained by the fact that the calibration data did not fully cover the range of reaction states occurring in the transient phase.

The largest contribution to the observed uncertainty originates from the reference data (low-field NMR, 43 MHz, Table 1), as is known from the validation of the presented NMR module by high-field NMR spectroscopy at 500 MHz [19]. The RMSE values obtained during this validation campaign represent the deviation of the low-field from the high-field NMR data and range from 12 mmol L^−1^ for Li-NDPA to 16 mmol L^−1^ for aniline. In fact, the RMSE values obtained from systematic cross-validation of NIR spectra are in the same order of magnitude suggesting a successful calibration transfer between both methods.

## Conclusion

An explosion-proof online NMR sensor with sampling rates of 15 s was used to monitor the chemical composition of the product stream of a metal-organic reaction in a pharmaceutical pilot plant. The applied modularized data evaluation approach requires only NMR spectra of the pure components as spectral model input. Absolute quantification was achieved in the pilot plant by one-point calibration based on a known raw material concentration. This method tremendously reduces set-up times of the NMR module. A quick adaption to new products can be realized by exchanging the spectral models according to the altered chemistry.

Concentration values provided by the online NMR sensor were utilized for iterative optimization of the plant performance. This approach has driven the process quickly to its economic optimum using a simple process model. The scheme adapted autonomously to a sudden change of feed composition which was not measured. In addition, the NMR sensor was successfully used as an online reference method for the calibration of a NIR spectrometer. Unstable lithiated intermediates, which could not be calibrated using conventional offline analytical methods like HPLC, were accessible using the online NMR sensor. Quantitative NMR spectroscopy carried out with compact instruments has the potential to substitute offline laboratory analysis for calibration purposes by delivering quantitative reference data as an online method. However, the accuracy of NIR predictions is limited by the accuracy of the NMR reference values.

It is to be expected that the accuracy of the NMR sensor prototype will increase due to current device developments focusing on increasing magnetic field strengths and improved line shape specifications. Online NMR spectroscopy extends the capabilities for measuring compositions to completely new application areas where existing technologies (NIR, Raman, UV/VIS, etc.) cannot be applied due to a lack of reference data. The online NMR sensor supports a very flexible operation of multi-product plants and model-based control already in the launching phase of new products.

## Electronic supplementary material

ESM 1 (PDF 4373 kb)

## Data Availability

The data reported in this paper are available in the article and the supplementary materials, as well as in a public data repository (10.5281/zenodo.1438233).
