# Supplementary material for: Flexible automation with compact NMR spectroscopy for continuous production of pharmaceuticals
Source: Anal Bioanal Chem. 2019 Mar 23;411(14):3037–46. doi: 10.1007/s00216-019-01752-y (PMC6526149; doi:10.1007/s00216-019-01752-y)
Supplement: Supplementary file 1 — (PDF 4373 kb) [file 216_2019_1752_MOESM1_ESM.pdf]

Analytical and Bioanalytical Chemistry

Electronic Supplementary Material

Flexible automation with compact NMR spectroscopy for continuous production of pharmaceuticals

Simon Kern, Lukas Wander, Klas Meyer, Svetlana Guhl, Anwesh Reddy Gottu Mukkula, Manuel Holtkamp, Malte Salge, Christoph Fleischer, Nils Weber, Rudibert King, Sebastian Engell, Andrea Paul, Manuel Pereira Remelhe, Michael Maiwald

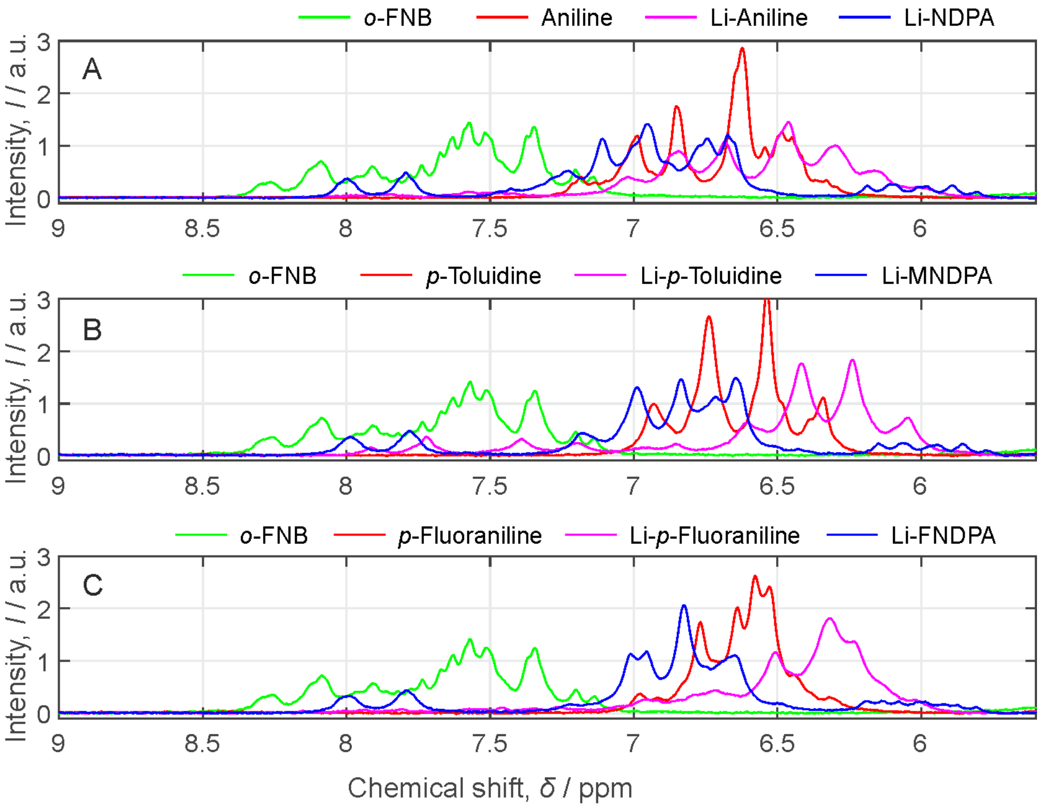

Fig. S1 Measured pure substance spectra of the reactants dissolved in THF, which occurred during the continuous synthesis of 2-nitrodiphenylamine (A, NDPA), 2-nitro-4'-methyldiphenylamine (B, MNDPA), and 2-nitro-4'-fluorodiphenylamine (C, FNDPA). ^{1}H spectra (43 MHz) were recorded as a single scan

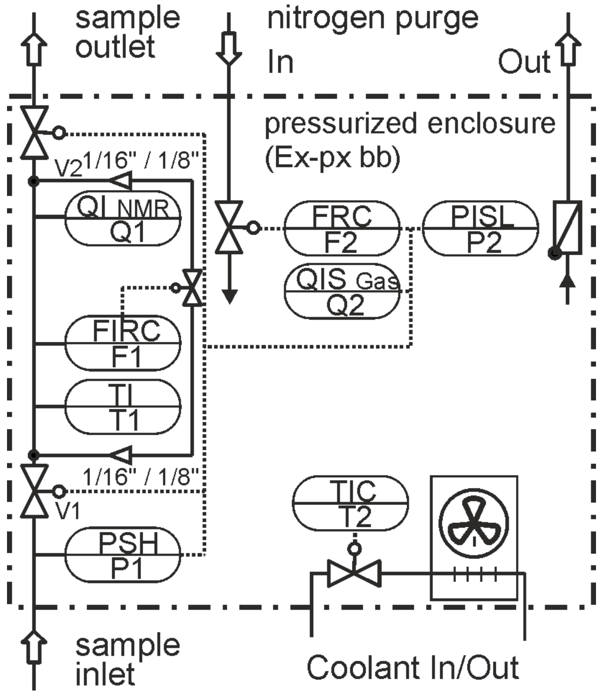

Fig. S2 P&ID of the online NMR module. Set-up with variable sample flow (>10 mL min^{-1}) whereby the flow through the NMR spectrometer is kept constant at 1.5 mL min^{-1}. The thresholds leading to the closing of safety valves V1 and V2 were implemented as follows: P1, 9.3 bar; P2, 0.89 mbar; Q1: 1000 ppm (vol/vol THF)

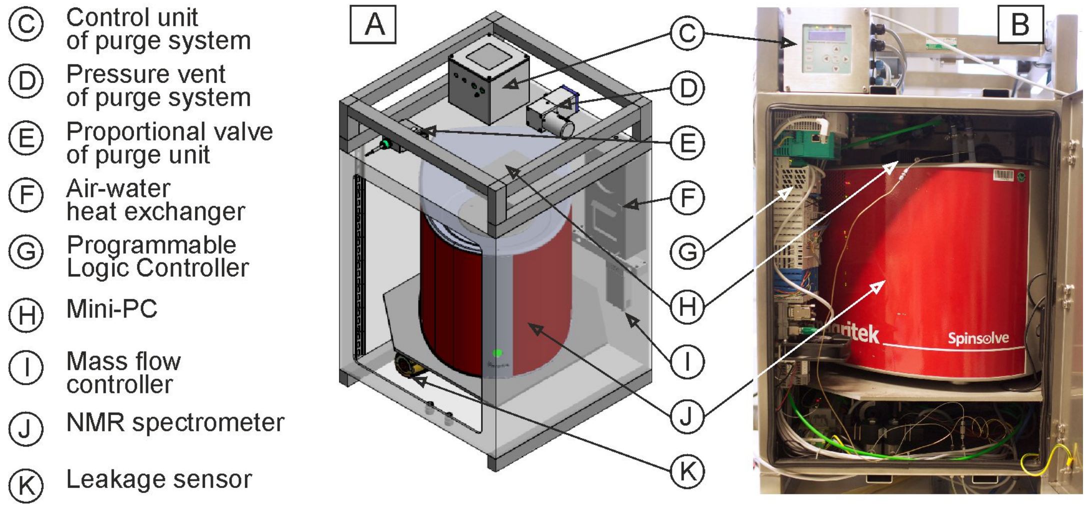

Fig. S3 Spatial arrangement of the components of the online NMR module, including a pressurized enclosure system during the design phase (A) and after system integration (B)

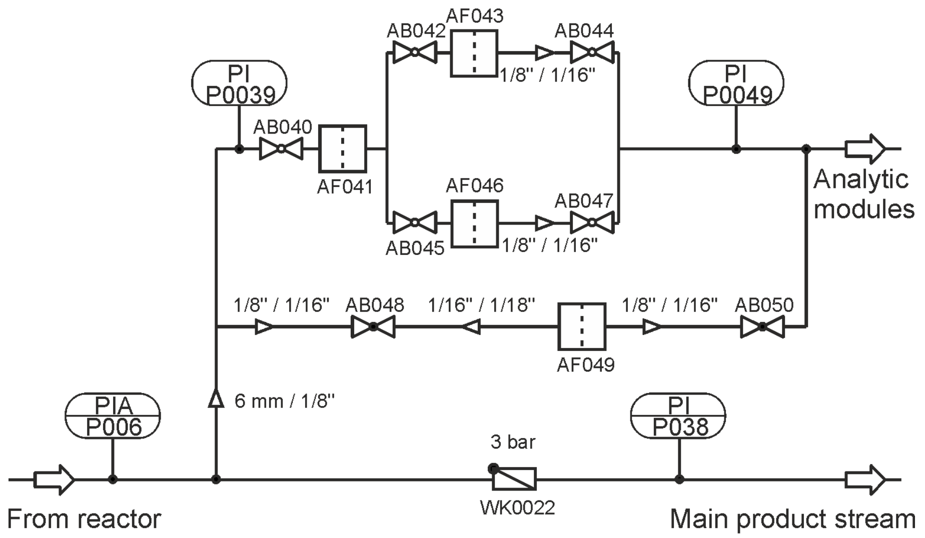

Fig. S4 P&ID of the filter section within the bypass to the analytic modules (NMR and NIR). A combination of 90 µm (AF041) and 60 µm (AF043 and Af046) stainless steel inline filters (Swagelok) has evolved as a robust arrangement to prevent clogging of the bypass. Due to the redundant design of the filter section, exchange of clogged filters during the production was possible

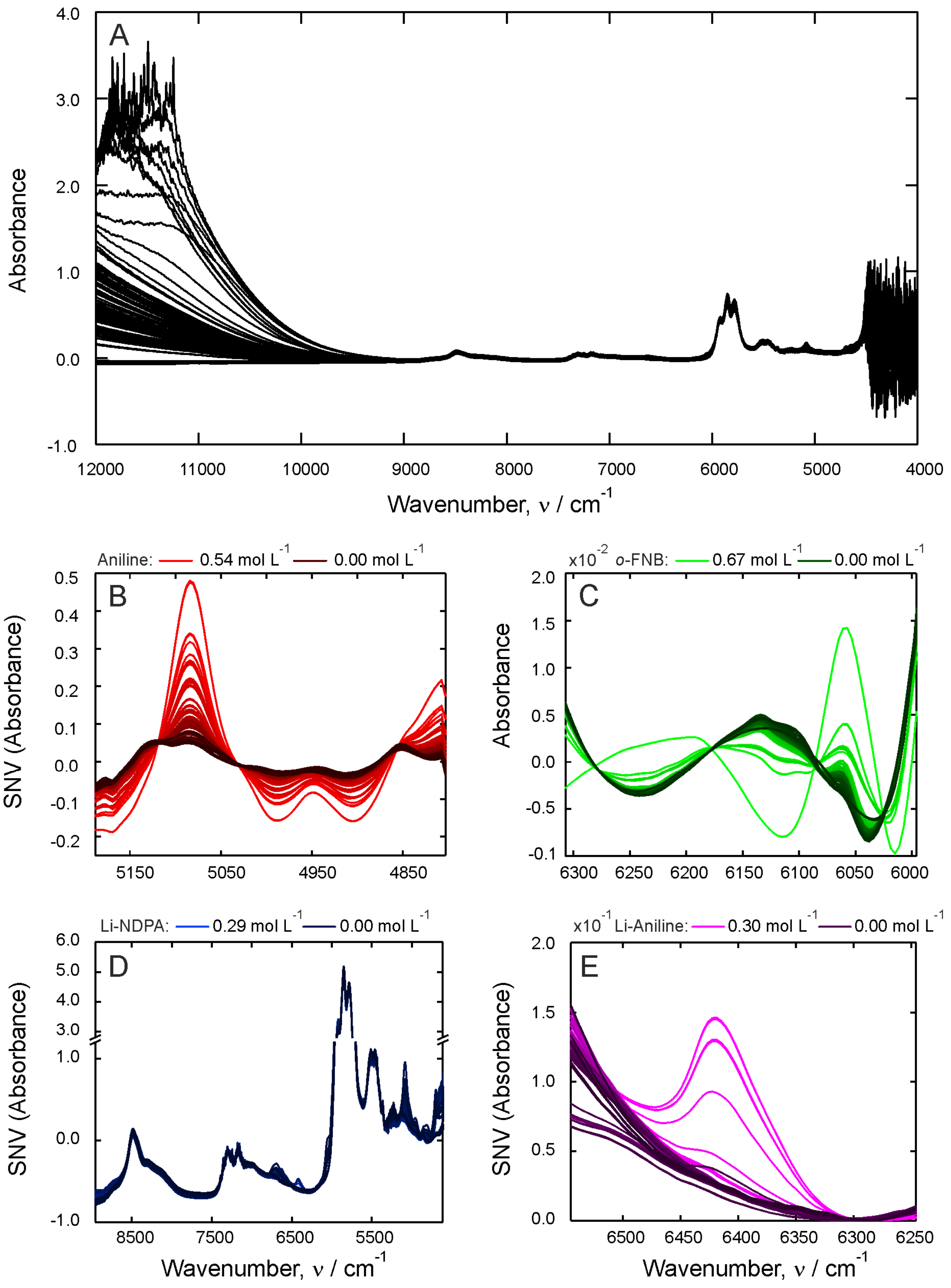

Fig. S5 Measured NIR spectra over the full spectral range of steady states during three test runs on days one two, and four comprising the calibration set (A). B–E shows pretreated spectra according to Table S1. Based on individually optimized spectral pretreatment strategies for the reactants aniline (B), *o*-FNB (C), the product Li-NDPA (D) and lithiated aniline (E), individual PLS1 models were developed using NMR concentration values as a reference

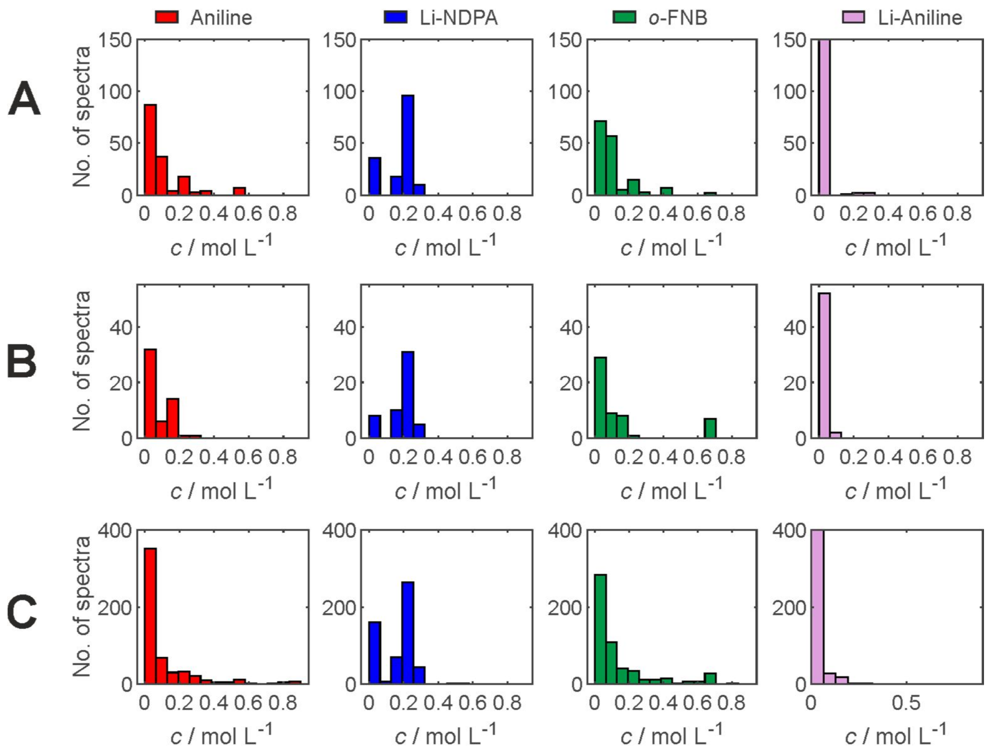

Fig. S6 Histograms of assigned reference values from low-field NMR data for multivariate analysis of NIR spectra. The data set was split into a calibration set (A), a validation set (B), and a test set (C)

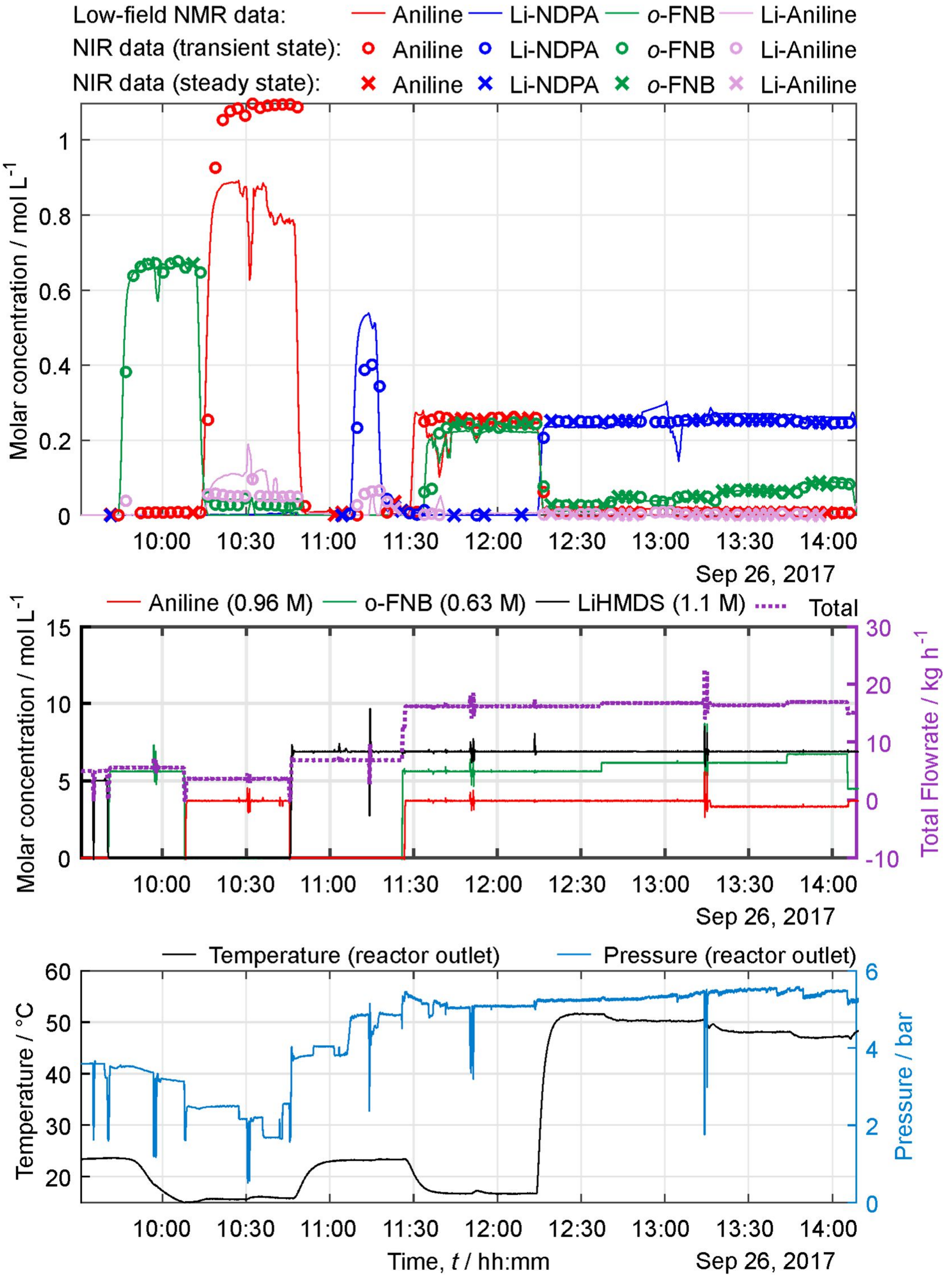

Fig. S7 Time course of relevant process data during continuous NDPA synthesis in the modular production plant (26.09.2017, production day 1, part A)

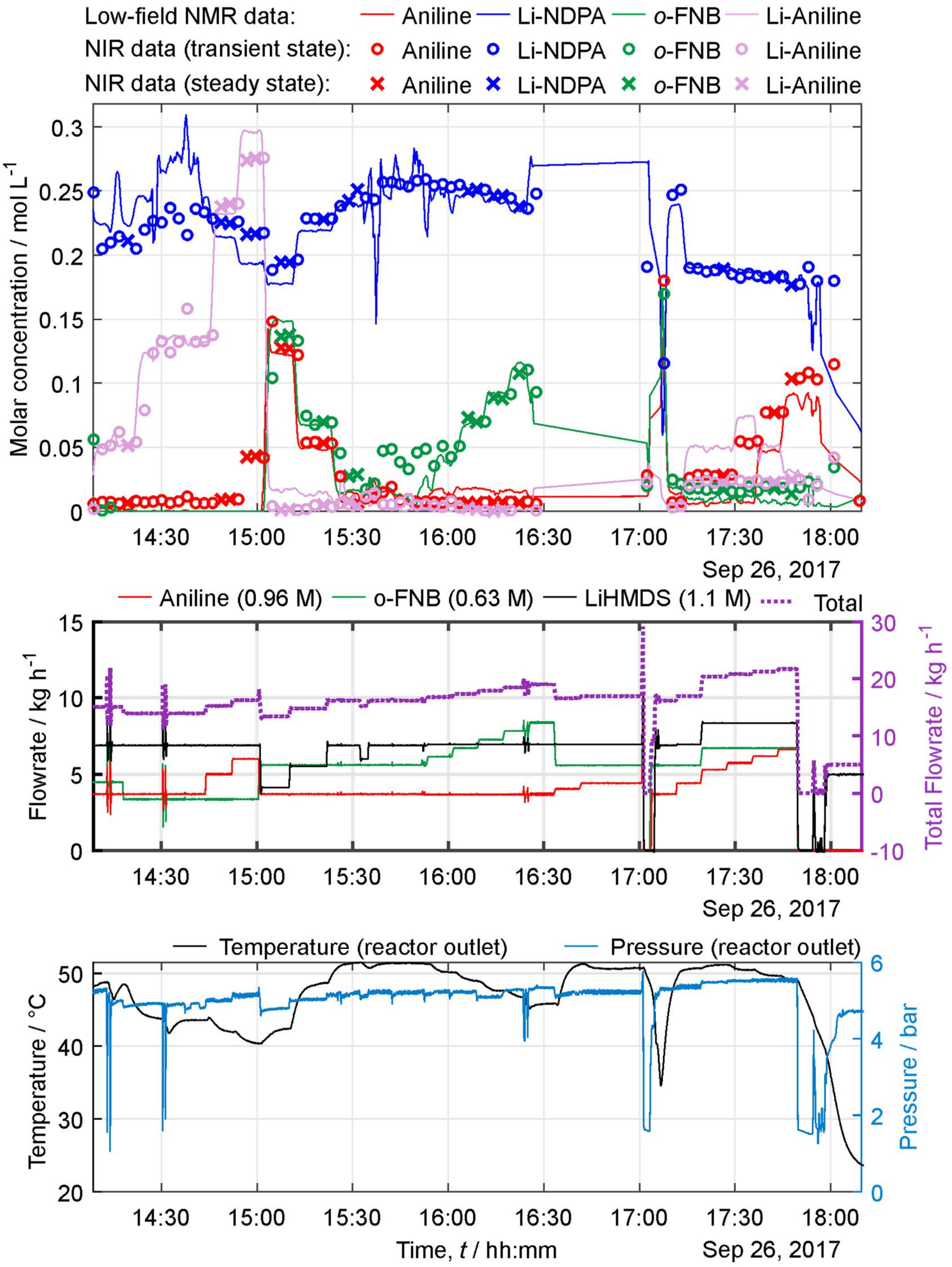

Fig. S8 Time course of relevant process data during continuous NDPA synthesis in the modular production plant (26.09.2017, production day 1, part B)

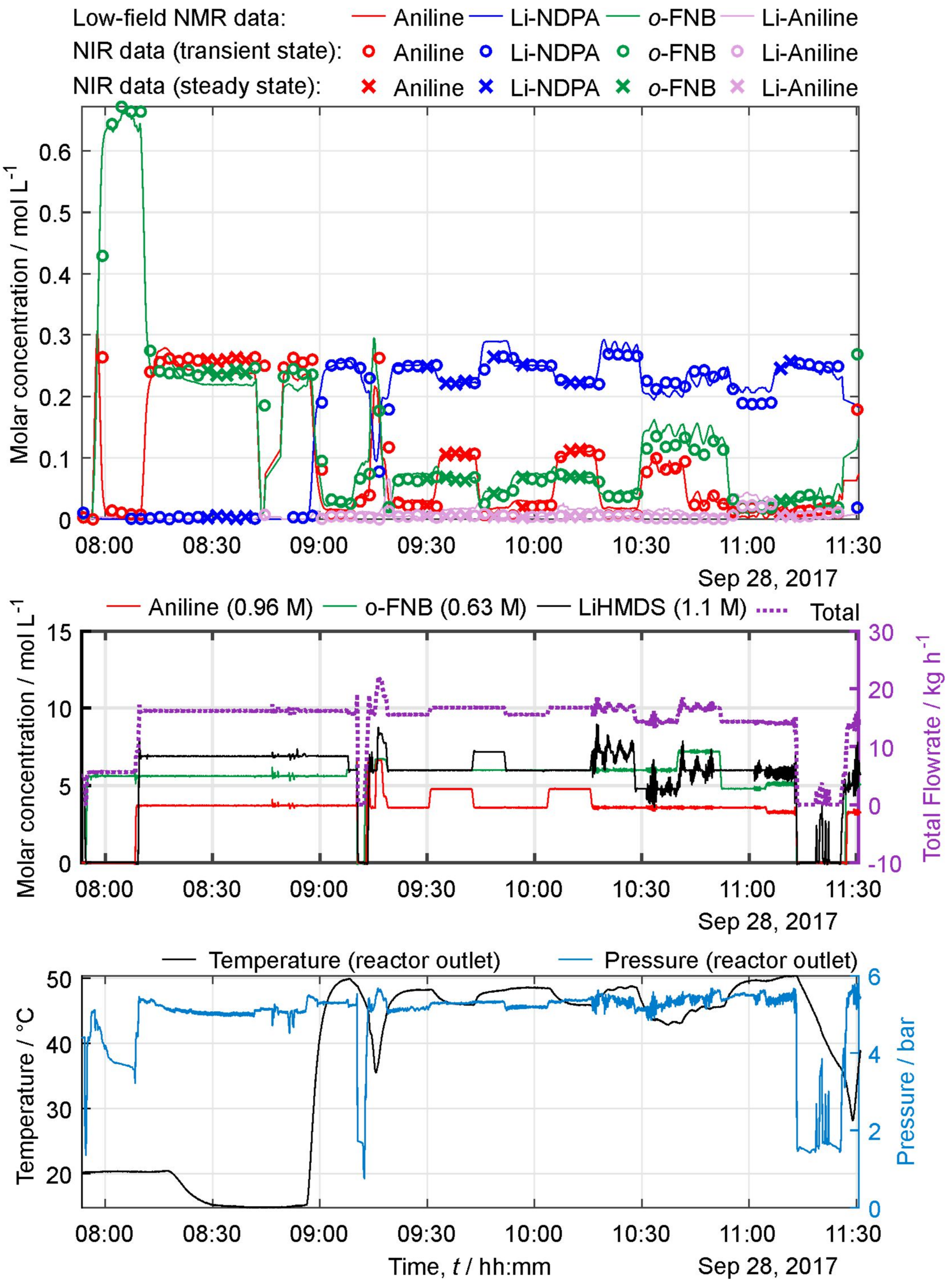

Fig. S9 Time course of relevant process data during continuous NDPA synthesis in the modular production plant (28.09.2017, production day 2, part A)

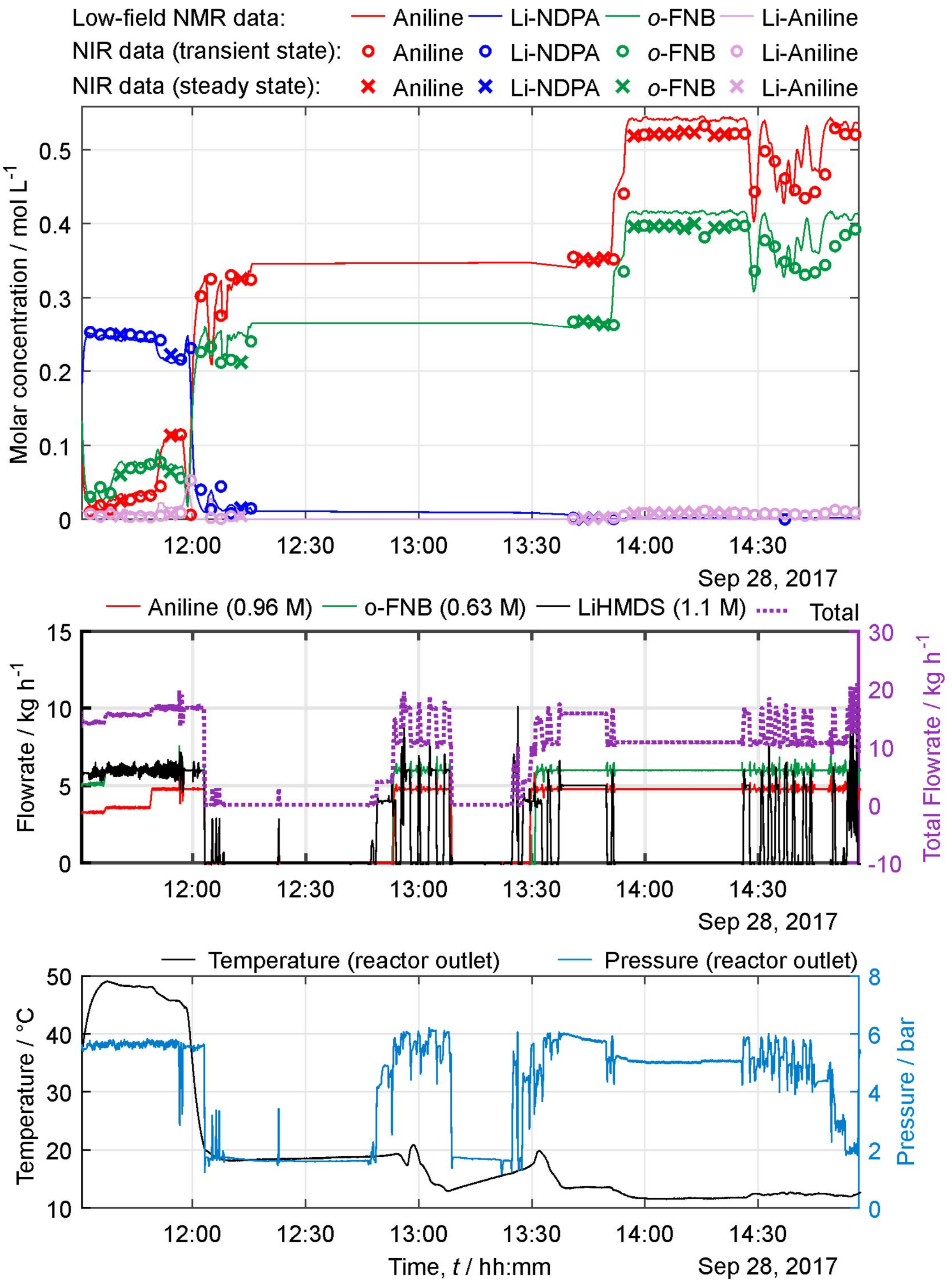

Fig. S10 Time course of relevant process data during continuous NDPA synthesis in the modular production plant (28.09.2017, production day 2, part B)

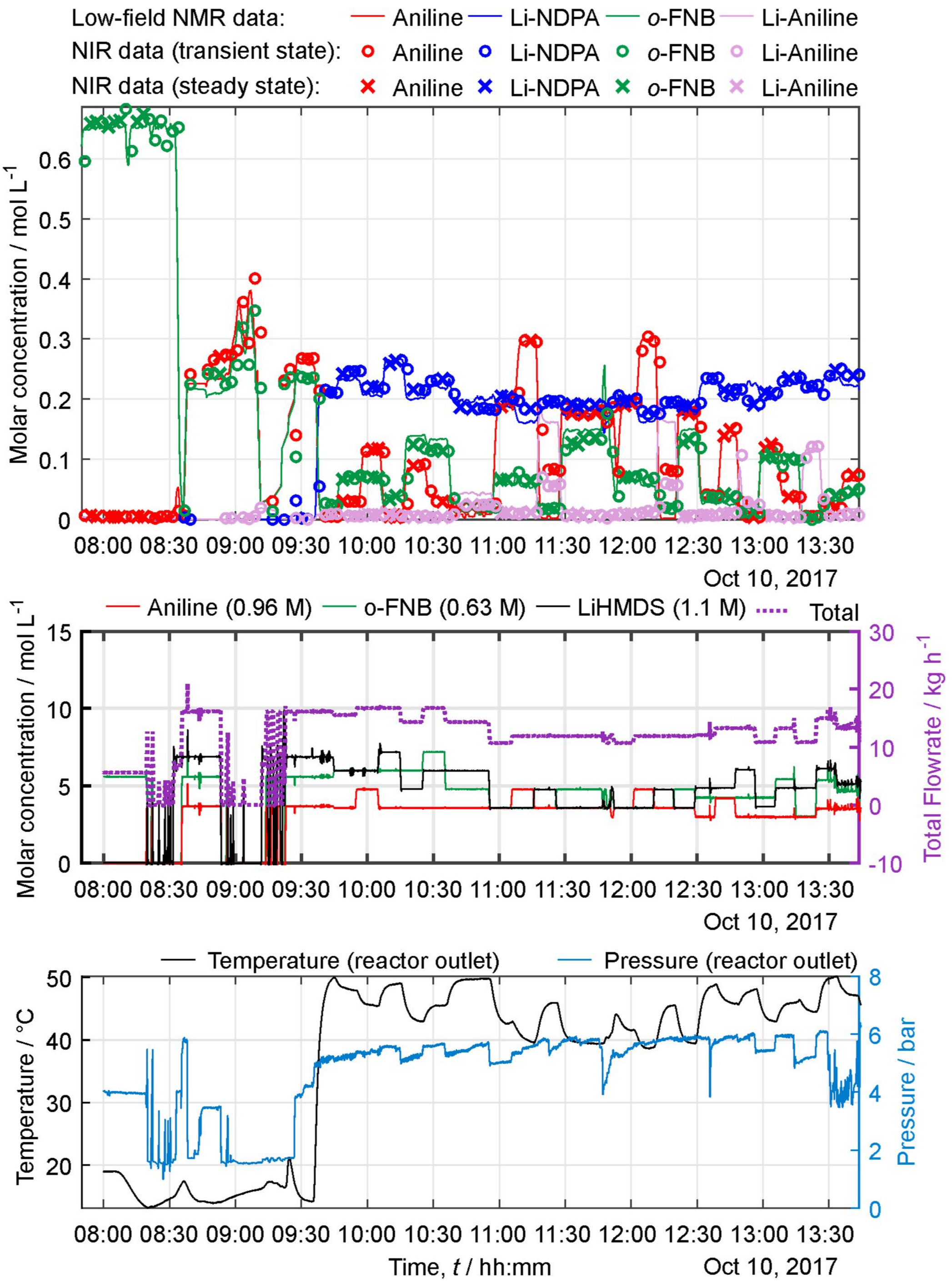

Fig. S11 Time course of relevant process data during continuous NDPA synthesis in the modular production plant (10.10.2017, production day 3, part A)

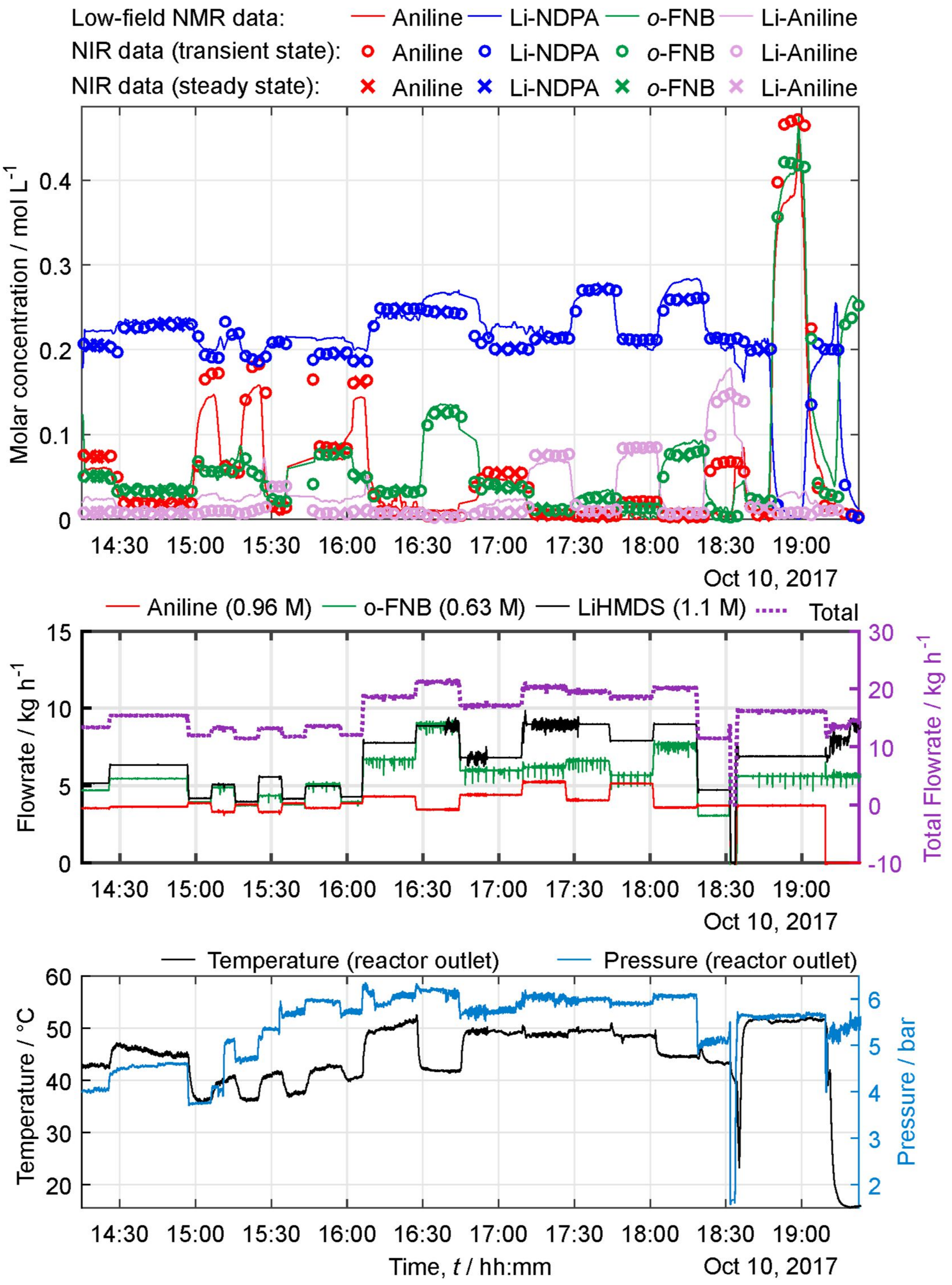

Fig. S12 Time course of relevant process data during continuous NDPA synthesis in the modular production plant (10.10.2017, production day 3, part B)

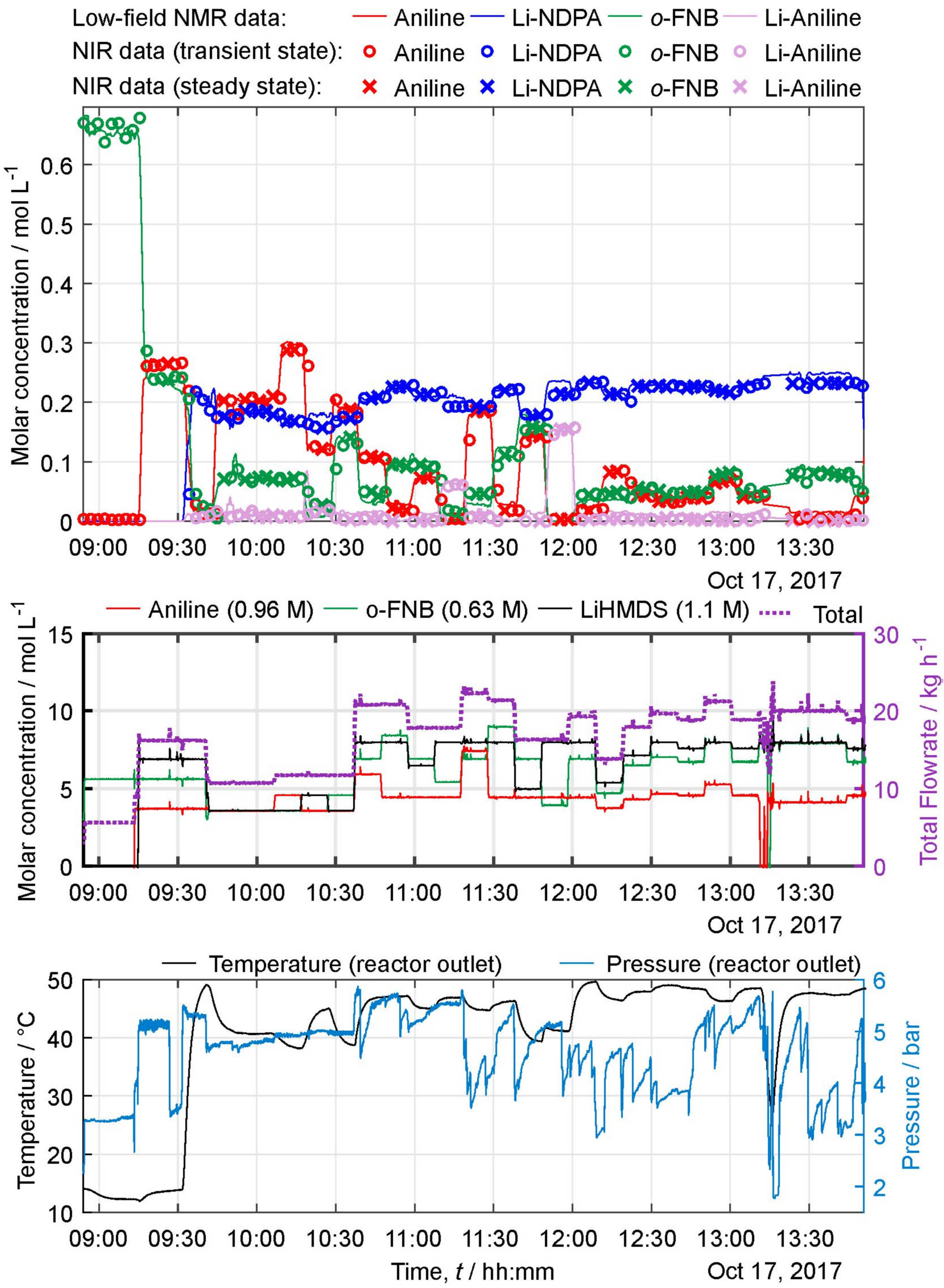

Fig. S13 Time course of relevant process data during continuous NDPA synthesis in the modular production plant (17.10.2017, production day 4, part A). Probing moves were performed between 9:15 h and 13:30 h. Subsequently the MAWQA algorithm was initialized

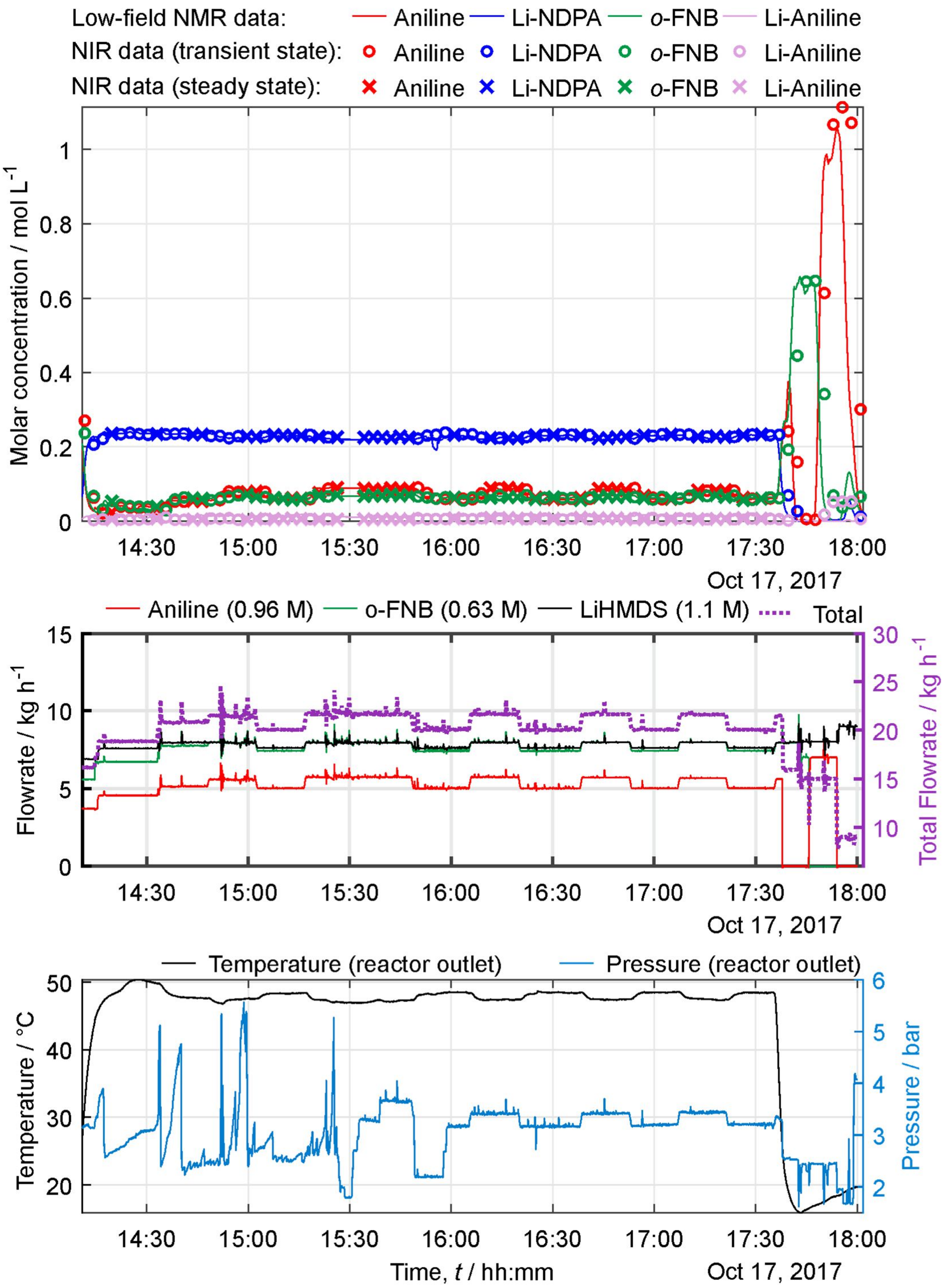

Fig. S14 Time course of relevant process data during continuous NDPA synthesis in the modular production plant (17.10.2017, production day 4, part B)

Table S1 Pretreatment of NIR spectra prior to PLSR applied in consecutive order

**Substance**	**Spectral range**	**Transformation**
Aniline	8957 cm^{-1}–4611 cm^{-1}	SNV
	5188 cm^{-1}–4803 cm^{-1}	Detrend, polynomial order 2
o-FNB	6307 cm^{-1}–5995 cm^{-1}	Detrend, polynomial order 3
Li-NDPA	8957 cm^{-1}–4611 cm^{-1}	SNV
Li-aniline	8957 cm^{-1}–4611 cm^{-1}	SNV
	6545 cm^{-1}–6245 cm^{-1}	Baseline correction (offset)
